# Perfect intrinsic squeezing at the superradiant phase transition critical point

**DOI:** 10.1038/s41598-023-29202-x

**Published:** 2023-02-13

**Authors:** Kenji Hayashida, Takuma Makihara, Nicolas Marquez Peraca, Diego Fallas Padilla, Han Pu, Junichiro Kono, Motoaki Bamba

**Affiliations:** 1 Department of Electrical and Computer Engineering, Rice University, Houston, TX 77005 USA; 2 Division of Applied Physics, Graduate School and Faculty of Engineering, Hokkaido University, Sapporo, Hokkaido 060-8628 Japan; 3 Department of Physics and Astronomy, Rice University, Houston, TX 77005 USA; 4 Department of Materials Science and Nano Engineering, Rice University, Houston, TX 77005 USA; 5 Department of Physics I, Kyoto University, Kitashirakawa Oiwake-cho, Sakyo-ku, Kyoto, 606-8502 Japan; 6 The Hakubi Center for Advanced Research, Kyoto University, Kyoto, 606-8501 Japan; 7 PRESTO, Japan Science and Technology Agency, Kawaguchi, 332-0012 Japan

**Keywords:** Quantum optics, Quantum mechanics, Phase transitions and critical phenomena

## Abstract

Some of the most exotic properties of the quantum vacuum are predicted in ultrastrongly coupled photon–atom systems; one such property is quantum squeezing leading to suppressed quantum fluctuations of photons and atoms. This squeezing is unique because (1) it is realized in the ground state of the system and does not require external driving, and (2) the squeezing can be perfect in the sense that quantum fluctuations of certain observables are completely suppressed. Specifically, we investigate the ground state of the Dicke model, which describes atoms collectively coupled to a single photonic mode, and we found that the photon–atom fluctuation vanishes at the onset of the superradiant phase transition in the thermodynamic limit of an infinite number of atoms. Moreover, when a finite number of atoms is considered, the variance of the fluctuation around the critical point asymptotically converges to zero, as the number of atoms is increased. In contrast to the squeezed states of flying photons obtained using standard generation protocols with external driving, the squeezing obtained in the ground state of the ultrastrongly coupled photon–atom systems is resilient against unpredictable noise.

## Introduction

**Figure 1:**
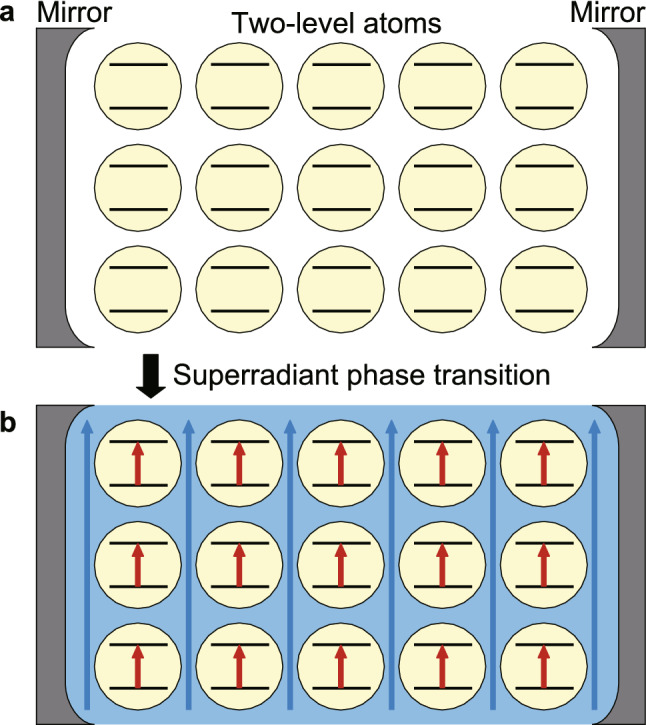
Sketches of system under investigation (Dicke model) and superradiant phase transition. The system consists of two-level atoms (yellow circles) collectively coupled with a single photonic field in a cavity composed with two mirrors. (**a**) In the normal phase, the expectation values of the photonic field (transverse electromagnetic field) and atomic field (electromagnetic polarization) are zero. (**b**) In the superradiant phase, the photonic and atomic fields (blue and red arrows, respectively, and order parameters) get static non-zero values spontaneously.

When photons strongly couple with an ensemble of atoms, there exists a threshold coupling strength above which a static photonic field (i.e., a transverse electromagnetic field) and a static atomic field (i.e., an electromagnetic polarization) are expected to appear spontaneously. This phenomenon, known as the superradiant phase transition (SRPT)^1,2^ depicted in Fig. 1, can occur at finite temperatures and at zero temperature. Since it was first proposed in 1973, the SRPT has attracted considerable attention from both experimental and theoretical researchers^3–10^.

In addition to experimental demonstrations of nonequilibrium SRPTs in atoms confined in optical cavities^3,4^, a superconducting-current version of the thermal-equilibrium SRPT was found theoretically in 2016^9^. A magnonic version was also confirmed in the magnetic material ErFeO_3_ in 2022^10^ based on a spin model that reproduces both experimental terahertz magnetospectroscopy^11^ and magnetization measurements^12^. In recent years, the possibility of realizing photonic SRPT under thermal equilibrium has also been debated actively for spatially-varying photonic modes coupled with interacting charged particles possessing the spin degree of freedom^5–8^. Those equilibrium SRPTs were discussed by mapping the specific systems into the Dicke model or its extended versions. The Dicke model represents a simple model where the SRPT can occurs^1,2^, and consists of an ensemble of two-level atoms collectively coupled to a single photonic field, as depicted in Fig. 1.

Although the finite-temperature SRPT is a classical phase transition given that it is driven by thermal fluctuations^13^ (in some studies ^3,14,15^, the SRPT realized by changing a system parameter is called a quantum SRPT when the term to be changed is not commutable with the rest of the Hamiltonian), quantum aspects of the SRPT at zero temperature have been investigated in terms of quantum chaos^14,15^, entanglement entropy^16^, and individual photonic and atomic squeezing^15–19^. In photonic (atomic) squeezing, the quantum fluctuation of the photonic (atomic) field is suppressed in one quadrature, whereas its conjugate fluctuation is enlarged while satisfying the Heisenberg uncertainty principle.

The critical (threshold) coupling strength required for realizing the SRPT exists in the ultrastrong or deep strong photon–atom coupling regime^20–22^, in which the photon–atom coupling strength (or vacuum Rabi splitting) is a considerable fraction of the bare photonic and atomic resonance frequency. It is known that ultrastrongly coupled light–matter systems exhibit so-called intrinsic squeezing^20,23–27^. Here, the *intrinsic* nature lies in the fact that squeezing exists in the ground state of the coupled light–matter system in thermal equilibrium without any external driving. This type of squeezing is in stark contrast to standard quantum squeezing, which is produced only in the presence of an external driving field. Note that intrinsic squeezing can occur even in the normal phase (i.e., zero expectation values of the photonic and atomic fields).

Critical quantum behavior, such as perfect spin squeezing^28–30^ and quantum Fisher information divergence^30^, is expected to emerge *intrinsically* at the onset of the SRPT, as the entanglement entropy is known to diverge at the SRPT critical point^16^. Although a universal behavior of thermal and quantum fluctuations around the SRPT critical point has been investigated recently in a generalized Dicke model with a finite number of atoms at finite temperatures^19^, critical behaviors of quantum fluctuations have not been reported even in the limit of an infinite number of atoms (thermodynamic limit) at zero temperature.

In this study, we show that *perfect* squeezing, where quantum fluctuations completely vanish in one quadrature, can be obtained in an appropriate photon–atom two-mode basis at the onset of the SRPT in the Dicke model under the thermodynamic limit. Unlike traditional squeezing generation in dynamic and nonequilibrium systems^31,32^, this squeezing is *intrinsic*, i.e., it emerges in equilibrium. These facts imply that the SRPT can provide high squeezing stably in equilibrium situations. This might open a new avenue for quantum sensing^33^ and continuous-variable quantum information technologies^34,35^, because the squeezing in equilibrium is obtained in the most stable state of systems and intrinsically robust against decoherence.

## Results

### Model

We consider the isotropic Dicke model^36^, whose Hamiltonian is given by

1 $$\frac{{{\hat{\mathcal{H}}}_{{{\text{Dicke}}}} }}{\hbar } = \omega_{a} \hat{a}^{\dagger } \hat{a} + \omega_{b} \left( {\hat{S}_{z} + \frac{N}{2}} \right) + \frac{2g}{{\sqrt N }}\left( {\hat{a}^{\dagger } + \hat{a}} \right)\hat{S}_{x} .$$

Here, $\widehat{a}$ is the annihilation operator of a photon with resonance frequency ${\omega }_{a}$. The first term corresponds to the energy of the photons. ${\widehat{S}}_{x,y,z}$ are the collective spin $\frac{N}{2}$ operators representing an ensemble of $N$ two-level atoms with the transition frequency ${\omega }_{b}$. The second term in Eq. (1) corresponds to the energy of the atoms. The last term represents the coupling between the photons and the atomic ensemble; $g$ is the coupling strength and it is assumed to be real and positive for simplicity. In terms of the lowering and raising operators ${\widehat{S}}_{\pm }\equiv {\widehat{S}}_{x}\pm \mathrm{i}{\widehat{S}}_{y}=\{{\widehat{S}}_{\mp }{\}}^{\dagger}$, the last term (i.e., the photon–atom coupling term) in Eq. (1) can be rewritten as $2g({\widehat{a}}^{\dagger}+\widehat{a}){\widehat{S}}_{x}/\sqrt{N}=g({\widehat{a}}^{\dagger}+\widehat{a})({\widehat{S}}_{+}+{\widehat{S}}_{-})/\sqrt{N}.$ Among these four terms, ${\widehat{a}}^{\dagger}{\widehat{S}}_{-}$ and ${\widehat{S}}_{+}\widehat{a}$ are co-rotating terms that are responsible for the vacuum Rabi splitting, whereas ${\widehat{a}}^{\dagger}{\widehat{S}}_{+}$ and $\widehat{a}{\widehat{S}}_{-}$ are counter-rotating terms that are responsible for the vacuum Bloch–Siegert shift^37,38^. As discussed later, these counter-rotating terms are responsible for the two-mode squeezing^20,23–27^.

### Intrinsic squeezing for finite numbers of atoms

We first numerically analyze the wavefunction of the ground state $\left|0\right.\rangle$ of the Dicke model, Eq. (1), for finite number $N$ of atoms. In numerical calculations, we rewrite ${\widehat{H}}_{\mathrm{Dicke}}$ as a matrix on the basis of ${\left|n\right.\rangle }_{a}{\left|\frac{N}{2},m\right.\rangle }_{S}$, where ${\left|n\right.\rangle }_{a}$ is the photonic Fock state with $n=0, 1, 2,\dots$ and ${\left|\frac{N}{2},m\right.\rangle }_{S}$ represents the atomic state (spin $\frac{N}{2}$ state) with $m=0, \pm 1, \pm 2,\pm \frac{N}{2}$.

For obtaining the wavefunction, we first calculate the Q function^31^ ${Q}_{ab}\left(\alpha ,\beta \right)={\left|\langle \alpha ,\beta |0\rangle \right|}^{2}/{\pi }^{2}$ on the basis of the coherent states $\left|\alpha ,\beta \right.\rangle$ with photonic amplitude $\alpha \in {\mathbb{C}}$ and atomic one $\beta \in {\mathbb{C}}$, whose definition is shown in “Methods”. We then calculate the Wigner function $W\left(\alpha ,\beta \right)$ by transforming ${Q}_{ab}\left(\alpha ,\beta \right)$ along the $(\alpha -\beta )/\sqrt{2}$ axis (see details in “Methods”).

**Figure 2:**
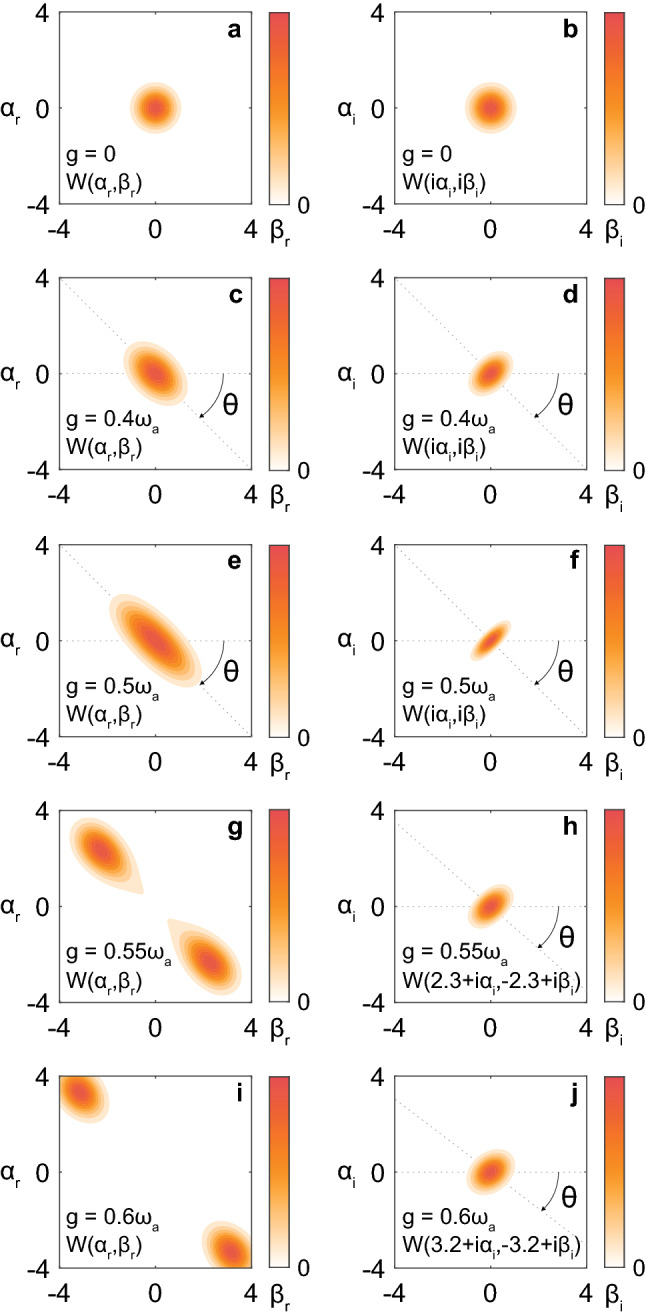
Wavefunctions $W\left(\alpha ,\beta \right)$ of the ground states of the Dicke model for $N={2}^{6}=64$ and ${\omega }_{b}={\omega }_{a}$. The photon–atom coupling strength is (**a, b**) $g=0$, (**c, d**) $g=0.4{\omega }_{a}$, (**e, f**) $g=0.5{\omega }_{a}$, (**g, h**) $g=0.55{\omega }_{a}$, and (**i, j**) $g=0.6{\omega }_{a}$. Panels (**a, c, e, g, i**) show $W({\alpha }_{\mathrm{r}},{\beta }_{\mathrm{r}})$ and (**b, d, f**) show $W(\mathrm{i}{\alpha }_{\mathrm{i}},\mathrm{i}{\beta }_{\mathrm{i}})$. Panels (**h**) and (**j**) show $W(2.3+\mathrm{i}{\alpha }_{\mathrm{i}},-2.3+\mathrm{i}{\beta }_{\mathrm{i}})$ and $W(3.2+\mathrm{i}{\alpha }_{\mathrm{i}},-3.2+\mathrm{i}{\beta }_{\mathrm{i}})$, where $\pm 2.3$ and $\pm 3.2$ are the peak positions at Panels g and i, respectively. For $0<g\le 0.5{\omega }_{a}$, $W\left(\alpha ,\beta \right)$ is anti-squeezed and squeezed in the ${\alpha }_{\mathrm{r}}-{\beta }_{\mathrm{r}}$ and ${\alpha }_{\mathrm{i}}-{\beta }_{\mathrm{i}}$ planes, respectively, along the direction of $\theta =-0.25\pi$. For $g=0.55{\omega }_{a}$ and $0.6{\omega }_{a}$, $W\left(\alpha ,\beta \right)$ gets two peaks in the ${\alpha }_{\mathrm{r}}-{\beta }_{\mathrm{r}}$ plane and becomes less squeezed than for $g=0.5{\omega }_{a}$ in the ${\alpha }_{\mathrm{i}}-{\beta }_{\mathrm{i}}$ plane. Parameters in the numerical calculations are shown in “Methods”. $W\left(\alpha ,\beta \right)$ is normalized to each maximum value.

Figure 2 shows the wavefunctions $W\left(\alpha ,\beta \right)$ of the ground state $\left|0\right.\rangle$ of the Dicke model for $N={2}^{6}=64$ and ${\omega }_{b}={\omega }_{a}$, which were chosen just as an example and for simplicity. Here, ${\omega }_{b}={\omega }_{a}$ means that the atomic and photonic resonance frequencies are equal, i.e., zero detuning. We have numerically confirmed that the results in Fig. 2 keep the same tendency in detuned cases (${\omega }_{b}\ne {\omega }_{a}$). The photon–atom coupling strength was set to (a,b) $g=0$, (c,d) $g=0.4{\omega }_{a}$, (e,f) $g=0.5{\omega }_{a}$, (g,h) $g=0.55{\omega }_{a}$, and (i,j) $g=0.6{\omega }_{a}$. Here, $g=0$ means no coupling, and $g=0.5{\omega }_{a}$ corresponds to the critical coupling strength of the SRPT in the thermodynamic limit^1,2^.

Figure 2a, b show $W({\alpha }_{\mathrm{r}},{\beta }_{\mathrm{r}})$ and $W(\mathrm{i}{\alpha }_{\mathrm{i}},\mathrm{i}{\beta }_{\mathrm{i}})$, respectively, for $g=0$ (${\alpha }_{\mathrm{r},\mathrm{i}},{\beta }_{\mathrm{r},\mathrm{i}}\in {\mathbb{R}}$). We find that the wavefunction is localized at the origin $\alpha =\beta =0$, and the peak broadening (corresponding to quantum fluctuations) is isotropic both in the ${\alpha }_{\mathrm{r}}-{\beta }_{\mathrm{r}}$ and ${\alpha }_{\mathrm{i}}-{\beta }_{\mathrm{i}}$ planes, signature of the ground state not being squeezed.

By increasing the coupling strength $g$, as seen in Fig. 2c ($g=0.4{\omega }_{a}$) and Fig. 2e ($g=0.5{\omega }_{a}$), the peak is getting broader (quantum fluctuation is getting anti-squeezed) along the $({\alpha }_{\mathrm{r}}-{\beta }_{\mathrm{r}})/\sqrt{2}$ axis $(\theta =-0.25\pi )$. At the same time, as seen in Fig. 2d ($g=0.4{\omega }_{a}$) and Fig. 2f ($g=0.5{\omega }_{a}$), the peak is getting narrower (quantum fluctuation is getting squeezed) along the $({\alpha }_{\mathrm{i}}-{\beta }_{\mathrm{i}})/\sqrt{2}$ axis $(\theta =-0.25\pi )$.

Here, let us suppose that ${\alpha }_{\mathrm{r}}$ and ${\alpha }_{\mathrm{i}}$ correspond to the normalized (dimensionless) electric (displacement) field $D$ and vector potential $A$, respectively, and ${\beta }_{\mathrm{r}}$ and ${\beta }_{\mathrm{i}}$ correspond to the normalized electric polarization $P$ and current $J$, respectively. In this case, $({\alpha }_{\mathrm{r}}-{\beta }_{\mathrm{r}})/\sqrt{2}$ corresponds to the difference between $D$ and $P$, and $({\alpha }_{\mathrm{i}}-{\beta }_{\mathrm{i}})/\sqrt{2}$ corresponds to the difference between $A$ and $J$. Figure 2a–f imply that, by increasing $g$ from 0 (no coupling), the quantum fluctuations in $D$–$P$ difference and $A$–$J$ one are getting anti-squeezed and squeezed, respectively. In other words, the quantum fluctuations of $A$ and $J$ are getting synchronized unlike those in the no-coupling case ($g=0$).

For larger $g$, as seen in Fig. 2g ($g=0.55{\omega }_{a}$) and Fig. 2i ($g=0.6{\omega }_{a}$), $W({\alpha }_{\mathrm{r}},{\beta }_{\mathrm{r}})$ gets two peaks at ${\alpha }_{\mathrm{r}}=\pm \overline{\alpha }$ and ${\beta }_{\mathrm{r}}=\mp \overline{\beta }$ ($\overline{\alpha },\overline{\beta }\in {\mathbb{R}}$). This means that the system energy is minimized around $\left|\pm \overline{\alpha },\mp \overline{\beta }\right.\rangle$, by which the photon–atom coupling term, the last term in Eq. (1), decreases the system energy approximately by $4g\overline{\alpha }\overline{\beta }\sqrt{1-{\overline{\beta }}^{2}/N}$ (see details in next subsection). When this energetical benefit is larger than the energetical demerit ${\omega }_{a}{\overline{\alpha }}^{2}+{\omega }_{b}{\overline{\beta }}^{2}$ [energy required for creating photons and exciting atoms; derived from the first and second terms in Eq. (1)], the peak in the ground-state wavefunction $W({\alpha }_{\mathrm{r}},{\beta }_{\mathrm{r}})$ is displaced from the origin (system energy is minimized around $\left|\pm \overline{\alpha },\mp \overline{\beta }\right.\rangle$).

However, because of the parity symmetry^14,15^ of the Dicke model, Eq. (1), the true ground state should include a superposition of the two states $\left|\pm \overline{\alpha },\mp \overline{\beta }\right.\rangle$ in the case of finite $N$. In the thermodynamic limit ($N\to \infty$), the parity symmetry is spontaneously broken, and the ground state becomes well approximated by one of $\left|\pm \overline{\alpha },\mp \overline{\beta }\right.\rangle$. Thus, the photonic and atomic fields spontaneously get no-zero order parameters $\left.\langle 0\right|\widehat{a}\left|0\right.\rangle \approx \pm \overline{\alpha }\in {\mathbb{R}}$ (corresponding to $D$) and $\left.\langle 0\right|{\widehat{S}}_{x}\left|0\right.\rangle \approx \mp \overline{\beta }\sqrt{1-{\overline{\beta }}^{2}/N}$ (corresponding to $P$), respectively. This is the basic picture of the SRPT. The SRPT critical coupling strength is $g=\sqrt{{\omega }_{a}{\omega }_{b}}/2$ (see details in next subsection), which corresponds to Fig. 2e, f ($g=0.5{\omega }_{a})$ in the zero-detuning case (${\omega }_{b}={\omega }_{a}$).

Figure 2h, j show $W(2.3+\mathrm{i}{\alpha }_{\mathrm{i}},-2.3+\mathrm{i}{\beta }_{\mathrm{i}})$ for $g=0.55{\omega }_{a}$ and $W\left(3.2+\mathrm{i}{\alpha }_{\mathrm{i}},-3.2+\mathrm{i}{\beta }_{\mathrm{i}}\right)$ for $g=0.6{\omega }_{a}$, where the wavefunction is maximized at $\overline{\alpha }\approx \overline{\beta }\approx 2.3$ and $\approx 3.2$ as seen in Fig. 2g, i, respectively. We can find that, by increasing $g$ from $0.5{\omega }_{a}$, the peak is getting broader (less squeezed), i.e., going back to be isotropic, in the ${\alpha }_{\mathrm{i}}-{\beta }_{\mathrm{i}}$ plane, whereas the squeezing direction $\theta$ is shifted from $-0.25\pi$. At the same time, as seen in Fig. 2g, i, each peak is getting narrower (less anti-squeezed), i.e., going back to be isotropic, in the ${\alpha }_{\mathrm{r}}-{\beta }_{\mathrm{r}}$ plane. In the limit of $g\gg \sqrt{{\omega }_{a}{\omega }_{b}}/2$, the ground state becomes well approximated by a classical state $\left|\pm \overline{\alpha },\mp \overline{\beta }\right.\rangle$ ^2,13–15^, i.e., each peak goes back to be isotropic both in the ${\alpha }_{\mathrm{r}}-{\beta }_{\mathrm{r}}$ and ${\alpha }_{\mathrm{i}}-{\beta }_{\mathrm{i}}$ planes, whereas the true ground state should include their superposition for satisfying the parity symmetry. In summary, the degree of squeezing becomes maximal around the SRPT critical point, whereas we have considered the finite number of atoms in Fig. 2.

**Figure 3:**
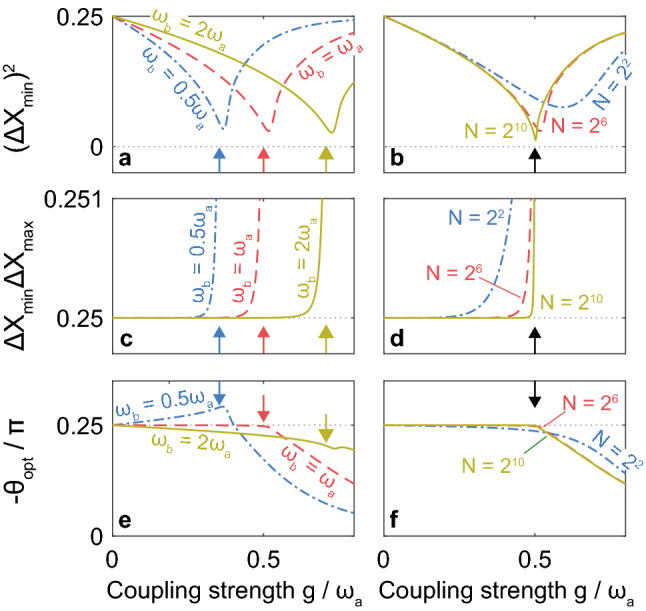
Minimum variance, deviation from standard quantum limit, and optimal angles as functions of coupling strength. (**a, b**) The minimum variance ${\left(\Delta {X}_{\mathrm{min}}\right)}^{2}$, Eq. (2), of the ground-state wavefunction $W({\mathrm{i}\alpha }_{\mathrm{i}},\mathrm{i}{\beta }_{\mathrm{i}})$ in the ${\alpha }_{\mathrm{i}}-{\beta }_{\mathrm{i}}$ plane, **c,d** the product of $\Delta {X}_{\mathrm{min}}$ and $\Delta {X}_{\mathrm{max}}$, Eq. (3), and (**e, f**) the optimal angle (squeezing angle in ${\alpha }_{\mathrm{i}}-{\beta }_{\mathrm{i}}$ plane) ${\theta }_{\mathrm{opt}}$ are plotted as functions of the photon–atom coupling strength $g$. In Panels (**a, c, e**), we assumed $N={2}^{6}=64$, ${\omega }_{b}=0.5{\omega }_{a}$ (blue dash-dotted line), ${\omega }_{b}={\omega }_{a}$ (red dashed line), and ${\omega }_{b}=2{\omega }_{a}$ (yellow line). In Panels (**b, d, f**), we assumed ${\omega }_{b}={\omega }_{a}$, $N={2}^{2}=4$ (blue dash-dotted line), $N={2}^{6}=64$ (red dashed line), and $N={2}^{10}=1024$ (yellow line). The arrows represent the critical point $g=\sqrt{{\omega }_{a}{\omega }_{b}}/2$ in the thermodynamic limit ($N\to \infty$). As seen in Panel a, ${\left(\Delta {X}_{\mathrm{min}}\right)}^{2}$ is minimized around the critical point. With the increase in $N$, the minimum point reaches the critical one and the minimum ${\left(\Delta {X}_{\mathrm{min}}\right)}^{2}$ monotonically decreases as seen in Panel b. The squeezing is almost ideal $\Delta {X}_{\mathrm{min}}\Delta {X}_{\mathrm{max}}\approx 0.25$ for $g\lesssim \sqrt{{\omega }_{a}{\omega }_{b}}/2$ and large enough $N$ as seen in Panels c and d. For $g>\sqrt{{\omega }_{a}{\omega }_{b}}/2$, $\Delta {X}_{\mathrm{min}}\Delta {X}_{\mathrm{max}}$ rapidly increases, because $W({\alpha }_{\mathrm{r}},{\beta }_{\mathrm{r}})$ gets two peaks as seen in Fig. 2g, i and $(\Delta {X}_{\mathrm{max}}{)}^{2}$ no longer corresponds to the broadening of each peak but represents the square of the distance between the two peaks. In contrast, $(\Delta {X}_{\mathrm{max}}{)}^{2}$ in Fig. 5c, g represents the broadening of each peak due to the spontaneous symmetry breaking (SRPT) in the thermodynamic limit ($N\to \infty$). Parameters in the numerical calculations are shown in “Methods”.

In order to better quantify the squeezing, in Fig. 3a, we plot the minimum variance ${\left(\Delta {X}_{\mathrm{min}}\right)}^{2}$ of the ground-state wavefunction $W({\mathrm{i}\alpha }_{\mathrm{i}},\mathrm{i}{\beta }_{\mathrm{i}})$ in the ${\alpha }_{\mathrm{i}}-{\beta }_{\mathrm{i}}$ plane as a function of $g$ for $N={2}^{4}=64$, ${\omega }_{b}=0.5{\omega }_{a}$ (blue dash-dotted line), ${\omega }_{b}={\omega }_{a}$ (red dashed line), and ${\omega }_{b}=2{\omega }_{a}$ (yellow line). We numerically searched for the optimal angle (squeezing angle) ${\theta }_{\mathrm{opt}}$ that provides the minimum variance ${\left(\Delta {X}_{\mathrm{min}}\right)}^{2}$, which was calculated by

2 $$(\Delta X_{{{\text{min}}}} )^{2} = \left\langle {\left. 0 \right|} \right.\left[ {\frac{{{\text{i}}\left( {\hat{a}^{\dag } - \hat{a}} \right)\cos \theta _{{{\text{opt}}}} + {\text{i}}\left( {\hat{b}^{\dag } - \hat{b}} \right)\sin \theta _{{{\text{opt}}}} }}{2}} \right]^{2} \left. {\left| 0 \right.} \right\rangle ,$$

i.e., by taking the expectation value of square of the operator $\left[\mathrm{i}\left({\widehat{a}}^{\dagger}-\widehat{a}\right)\mathrm{cos}{\theta }_{\mathrm{opt}}+\mathrm{i}\left({\widehat{b}}^{\dagger}-\widehat{b}\right)\mathrm{sin}{\theta }_{\mathrm{opt}}\right]/2$ corresponding to $\left({\alpha }_{\mathrm{i}}\mathrm{cos}{\theta }_{\mathrm{opt}}+{\beta }_{\mathrm{i}}\mathrm{sin}{\theta }_{\mathrm{opt}}\right)$, that is $\approx \left({\alpha }_{\mathrm{i}}-{\beta }_{\mathrm{i}}\right)/\sqrt{2}$ (${\theta }_{\mathrm{opt}}\approx -0.25\pi$) in the case of Fig. 2b, d, and f. Here, the atomic annihilation operator $\widehat{b}$ is defined in “Methods”. In Fig. 3a, we can find that ${\left(\Delta {X}_{\mathrm{min}}\right)}^{2}$ are minimized around the critical strength $g\approx \sqrt{{\omega }_{a}{\omega }_{b}}/2$ (indicated by arrows), whereas the actual minimum point is shifted due to finite $N$^14,15^.

Figure 3b shows ${\left(\Delta {X}_{\mathrm{min}}\right)}^{2}$ as a function of $g$ for ${\omega }_{b}={\omega }_{a}$ and $N={2}^{2}=4$ (blue dash-dotted line), ${2}^{6}=64$ (red dashed line), and ${2}^{10}=1024$ (yellow line). We can find that, with the increase in $N$, the minimum position shifts towards $g=\sqrt{{\omega }_{a}{\omega }_{b}}/2$, and the minimum ${\left(\Delta {X}_{\mathrm{min}}\right)}^{2}$ monotonically decreases.

In Fig. 3e, f, the optimal angle ${\theta }_{\mathrm{opt}}$ is plotted as a function of $g$. As we have seen in Fig. 2, ${\theta }_{\mathrm{opt}}\approx -0.25\pi$ for $g\lesssim 0.5{\omega }_{a}$ in the zero-detuning case (${\omega }_{b}={\omega }_{a}$). However, ${\theta }_{\mathrm{opt}}$ depends on ${\omega }_{b}/{\omega }_{a}$, $g/{\omega }_{a}$, and $N$, in general.

The anti-squeezing is quantified by the variance $(\Delta {X}_{\mathrm{max}}{)}^{2}$ of $W({\alpha }_{\mathrm{r}},{\beta }_{\mathrm{r}})$ in the ${\alpha }_{\mathrm{r}}-{\beta }_{\mathrm{r}}$ plane along the $\left({\alpha }_{\mathrm{r}}\mathrm{cos}{\theta }_{\mathrm{opt}}+{\beta }_{\mathrm{r}}\mathrm{sin}{\theta }_{\mathrm{opt}}\right)$ axis [broadening in Fig. 2a, c, e], which was evaluated by

3 $$(\Delta X_{{{\text{max}}}} )^{2} = \left\langle {\left. 0 \right|} \right.\left[ {\frac{{\left( {\hat{a}^{\dag } + \hat{a}} \right)\cos \theta _{{{\text{opt}}}} + \left( {\hat{b}^{\dag } + \hat{b}} \right)\sin \theta _{{{\text{opt}}}} }}{2}} \right]^{2} \left. {\left| 0 \right.} \right\rangle .$$

Figure 3c, d show $\Delta {X}_{\mathrm{min}}\Delta {X}_{\mathrm{max}}$ as a function of $g$. When the atomic subsystem is well approximated as a bosonic system, this quantity should satisfy the Heisenberg uncertainty principle $\Delta {X}_{\mathrm{min}}\Delta {X}_{\mathrm{max}}\ge 1/4$^31,32^, which is satisfied in all the cases in Fig. 3c, d. Further, we can find that $\Delta {X}_{\mathrm{min}}\Delta {X}_{\mathrm{max}}\approx 1/4$ is obtained for $g<\sqrt{{\omega }_{a}{\omega }_{b}}/2$, although $\Delta {X}_{\mathrm{min}}\Delta {X}_{\mathrm{max}}$ is slightly larger than 1/4 exactly at the critical strength $g=\sqrt{{\omega }_{a}{\omega }_{b}}/2$. When $g$ is larger than the critical value $\sqrt{{\omega }_{a}{\omega }_{b}}/2$, $\Delta {X}_{\mathrm{min}}\Delta {X}_{\mathrm{max}}$ rapidly increases from $1/4$. This is because $W({\alpha }_{\mathrm{r}},{\beta }_{\mathrm{r}})$ gets two peaks as seen in Fig. 2g, i, and $(\Delta {X}_{\mathrm{max}}{)}^{2}$ no longer corresponds to the broadening of each peak but represents the square of the distance between the two peaks^17^. In contrast, $(\Delta {X}_{\mathrm{max}}{)}^{2}$ in Fig. 5c, g represents the broadening of each peak under the spontaneous symmetry breaking (SRPT) in the thermodynamic limit ($N\to \infty$).

**Figure 4:**
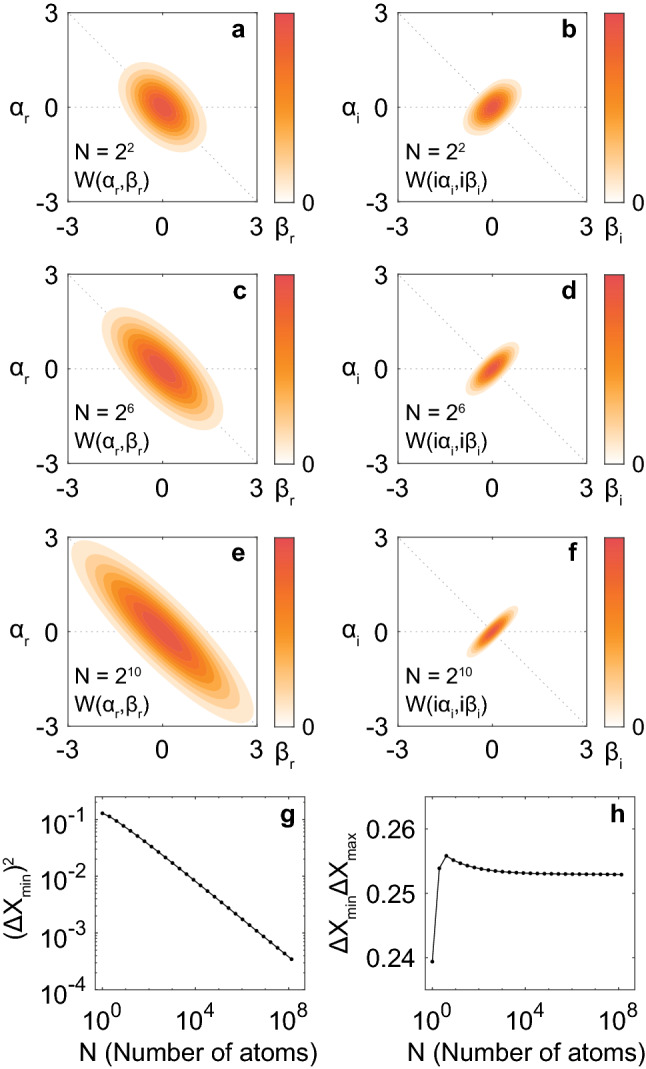
Wavefunctions $W\left(\alpha ,\beta \right)$ and the minimum variance in the ground states of the Dicke model for ${\omega }_{b}={\omega }_{a}$ and $g=0.5{\omega }_{a}$. (**a, b**) $N={2}^{2}=4$, (**c, d**) $N={2}^{6}=64$, and (**e, f**) $N={2}^{10}=1024$. Panels (**g**) and (**h**) show ${\left(\Delta {X}_{\mathrm{min}}\right)}^{2}$ and $\Delta {X}_{\mathrm{min}}\Delta {X}_{\mathrm{max}}$, respectively, as functions of $N$ for ${\omega }_{b}={\omega }_{a}$ and $g=0.5{\omega }_{a}$. With the increase in $N$, $W\left(\alpha ,\beta \right)$ is getting anti-squeezed and squeezed in the ${\alpha }_{\mathrm{r}}-{\beta }_{\mathrm{r}}$ and ${\alpha }_{\mathrm{i}}-{\beta }_{\mathrm{i}}$ planes, respectively, along the direction of $\theta =-0.25\pi$, and ${\left(\Delta {X}_{\mathrm{min}}\right)}^{2}$ monotonically decreases as seen in Panel g. Parameters in the numerical calculations are shown in “Methods”. $W\left(\alpha ,\beta \right)$ is normalized to each maximum value.

To see the tendency of squeezing with the increase in $N$ more in detail, in Fig. 4, we plot the ground-state wavefunctions $W(\alpha ,\beta )$ for ${\omega }_{b}={\omega }_{a}$, $g=0.5{\omega }_{a}$, and (a,b) $N={2}^{2}$, (c,d) $N={2}^{6}$, and (e,f) $N={2}^{10}$. By increasing $N$, we can find that $W({\alpha }_{\mathrm{r}},{\beta }_{\mathrm{r}})$ is getting broader along the $\left({\alpha }_{\mathrm{r}}-{\beta }_{\mathrm{r}}\right)/\sqrt{2}$ axis (Fig. 4a, c, and e) because of the $\sqrt{N}$-proportionality of the order parameters $\overline{\alpha }$ and $\overline{\beta }$^1,2^ (see also next subsection). On the other hand, $W({\mathrm{i}\alpha }_{\mathrm{i}},\mathrm{i}{\beta }_{\mathrm{i}})$ is getting narrower along the $\left({\alpha }_{\mathrm{i}}-{\beta }_{\mathrm{i}}\right)/\sqrt{2}$ axis (Fig. 4b, d, f).

Figure 4g shows the variance ${\left(\Delta {X}_{\mathrm{min}}\right)}^{2}$ along the $\left({\alpha }_{\mathrm{i}}-{\beta }_{\mathrm{i}}\right)/\sqrt{2}$ axis as a function of $N$ for ${\omega }_{b}={\omega }_{a}$ and $g=0.5{\omega }_{a}$. We numerically confirmed that ${\left(\Delta {X}_{\mathrm{min}}\right)}^{2}$ decreases monotonically with the increase in $N$ and reaches $3.5\times {10}^{-4}$ at $N={2}^{27}\approx {10}^{8}$.

Figure 4h shows $\Delta {X}_{\mathrm{min}}\Delta {X}_{\mathrm{max}}$ as a function of $N$. Although we got $\Delta {X}_{\mathrm{min}}\Delta {X}_{\mathrm{max}}<1/4$ for $N=1$ (atomic subsystem is not bosonic at all), we found $\Delta {X}_{\mathrm{min}}\Delta {X}_{\mathrm{max}}>1/4$ for larger $N$. As seen in Fig. 3d, $\Delta {X}_{\mathrm{min}}\Delta {X}_{\mathrm{max}}$ starts to increase rapidly around the critical point $g=0.5{\omega }_{a}$, thus we get $\Delta {X}_{\mathrm{min}}\Delta {X}_{\mathrm{max}}=0.253$ even at $N={2}^{27}\approx {10}^{8}$ in Fig. 4h. The asymptotic behavior of $\Delta {X}_{\mathrm{min}}\Delta {X}_{\mathrm{max}}$ is slower than that of ${\left(\Delta {X}_{\mathrm{min}}\right)}^{2}$. Regrettably, it was hard to discuss the $N$-dependence of $\Delta {X}_{\mathrm{min}}\Delta {X}_{\mathrm{max}}$ more in detail by our computational power.

As we have seen above, when the photon–atom coupling term is represented as $2g({\widehat{a}}^{\dagger}+\widehat{a}){\widehat{S}}_{x}/\sqrt{N}$ as in Eq. (1), the ground-state wavefunction of the Dicke model gets two peaks at $\left|\pm \overline{\alpha },\mp \overline{\beta }\right.\rangle$ for $g\gtrsim \sqrt{{\omega }_{a}{\omega }_{b}}/2$, and the best squeezing is obtained around the critical point $g=\sqrt{{\omega }_{a}{\omega }_{b}}/2$ along a certain direction in the ${\alpha }_{\mathrm{i}}-{\beta }_{\mathrm{i}}$ plane [along the $({\alpha }_{\mathrm{i}}-{\beta }_{\mathrm{i}})/\sqrt{2}$ axis in the zero-detuning case (${\omega }_{b}={\omega }_{a}$) as seen in Fig. 2]. These facts mean that, when ${\alpha }_{\mathrm{r}}$ and ${\alpha }_{\mathrm{i}}$ correspond to the normalized electric (displacement) field $D$ and vector potential $A$, respectively, and ${\beta }_{\mathrm{r}}$ and ${\beta }_{\mathrm{i}}$ correspond to the normalized electric polarization $P$ and current $J$, respectively, the ground state approximately becomes a superposition of two classical states with non-zero $\pm D$ and $\pm P$ for $g\gtrsim \sqrt{{\omega }_{a}{\omega }_{b}}/2$, and the best synchronization (squeezing) of the quantum fluctuations of $A$ and $J$ is obtained around the critical point $g=\sqrt{{\omega }_{a}{\omega }_{b}}/2$.

The $g$-dependence of the ground-state wavefunction $W({\alpha }_{\mathrm{r}},{\beta }_{\mathrm{r}})$ for real amplitudes ${\alpha }_{\mathrm{r}}$ and ${\beta }_{\mathrm{r}}$ has been discussed including the SRPT picture and the anti-squeezing by Emary and Brandes^15^. However, the squeezing seen in $W(\mathrm{i}{\alpha }_{\mathrm{i}},\mathrm{i}{\beta }_{\mathrm{i}})$ for imaginary amplitudes ${\alpha }_{\mathrm{i}}$ and ${\beta }_{\mathrm{i}}$ has been discussed just by focusing on one of them (basically imaginary photonic amplitude ${\alpha }_{\mathrm{i}}$)^15,17,19,39^. In Figs. 2 and 4, we found that the squeezing (narrowing) occurs along a certain direction in the ${\alpha }_{\mathrm{i}}-{\beta }_{\mathrm{i}}$ plane. It means that the ground state $\left|0\right.\rangle$ of the Dicke model should be described as a two-mode squeezed state as a one-mode squeezing discussed in Refs.^15,17,19,39^ cannot fully capture the squeezing features of $\left|0\right.\rangle$. By properly taking the two-mode basis $\left({\alpha }_{\mathrm{i}}-{\beta }_{\mathrm{i}}\right)/\sqrt{2}$ for $g=0.5{\omega }_{a}$ and ${\omega }_{b}={\omega }_{a}$, we found the monotonic decrease in $(\Delta {X}_{\mathrm{min}}{)}^{2}$ with the increase in $N$ in Fig. 4g.

### Perfect intrinsic squeezing in thermodynamic limit

In the previous subsection, we have numerically analyzed the squeezing of the ground state (intrinsic squeezing) of the Dicke model for finite numbers $N$ of atoms. In this subsection, we analyze the intrinsic squeezing in the thermodynamic limit ($N\to \infty$).

As the Dicke model is an effectively infinite-dimensional system^13^ in the thermodynamic limit, the SRPT can be analyzed under a mean-field framework^2,14,15,40,41^. Here, we follow the Holstein–Primakoff transformation approach^14,15,40,41^, which is suitable for zero-temperature analyses of the SRPT (spontaneous symmetry breaking). The spin operators are rewritten using a bosonic annihilation operator $\widehat{b}$ of the atomic collective excitations, as follows:

4 $$\hat{S}_{z} \to \hat{b}^{\dagger } \hat{b} - N/2,{ }\hat{S}_{ - } \to (N - \hat{b}^{\dagger } \hat{b})^{1/2} \hat{b}.$$

The appearance of the superradiant phase, where non-zero $\langle \widehat{a}\rangle =\overline{a }\sqrt{N}$ and $\langle \widehat{b}\rangle =-\overline{b }\sqrt{N}$ ($\overline{a },\overline{b}\in {\mathbb{R} }$) appear spontaneously, can be easily confirmed at zero temperature through the classical energy $\overline{\mathcal{H} }/(\hslash N)={\omega }_{a}{\overline{a} }^{2}+{\omega }_{b}{\overline{b} }^{2}-4g\overline{a }\overline{b }\sqrt{1-{\overline{b} }^{2}}$ obtained from Eq. (1). The zero-temperature classical state (the most stable state under this classical treatment, i.e., the state yielding the minimum of this classical energy) satisfies $\partial \mathcal{H}/\partial \overline{a }=\partial \mathcal{H}/\partial \overline{b }=0$, from which we obtain

5 $$\overline{a} = \frac{2g}{{\omega_{a} }}\overline{b}\sqrt {1 - \overline{b}^{2} } ,\quad \overline{b}^{2} = \left\{ {\begin{array}{*{20}l} {0,} \hfill & {g \le \sqrt {\omega_{a} \omega_{b} } /2} \hfill \\ {\frac{1}{2}\left( {1 - \frac{{\omega_{a} \omega_{b} }}{{4g^{2} }}} \right),} \hfill & {g > \sqrt {\omega_{a} \omega_{b} } /2} \hfill \\ \end{array} } \right..$$

**Figure 5:**
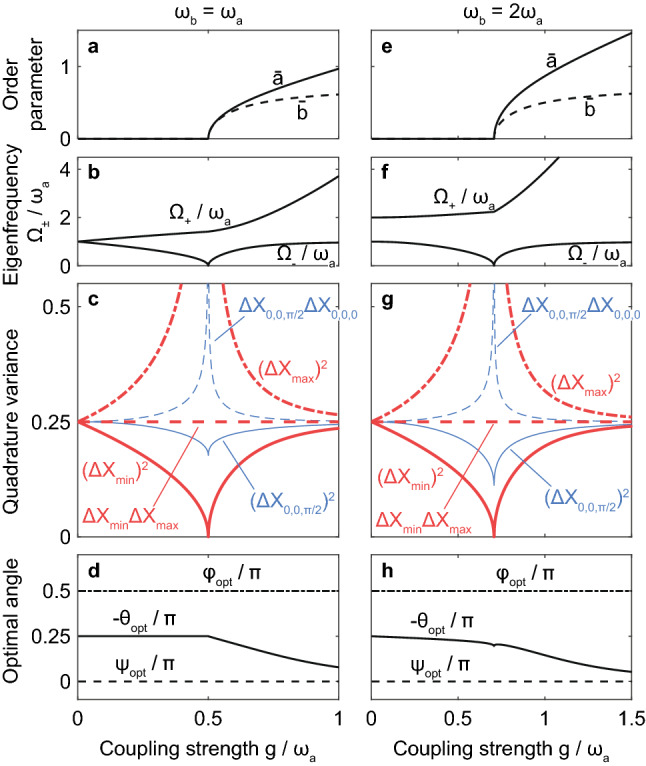
Numerical demonstration of perfect and intrinsic squeezing. (**a–d**) ${\omega }_{b}={\omega }_{a}$ and (**e–h**) ${\omega }_{b}=2{\omega }_{a}$. We plot, as a function of $g/{\omega }_{a}$, (**a, e**) order parameters $\overline{a }$ and $\overline{b }$; (**b, f**) eigenfrequencies ${\Omega }_{\pm }$; (**c, g**) quadrature variance; and (**d, h**) optimal angles ${\theta }_{\mathrm{opt}}$, ${\psi }_{\mathrm{opt}}$, and ${\varphi }_{\mathrm{opt}}$ that yield the minimum variance $(\Delta {X}_{\mathrm{min}}{)}^{2}$, indicated by the red bold solid line in Panels c and g. The minimum variance vanishes at the SRPT critical point ($g=\sqrt{{\omega }_{a}{\omega }_{b}}/2$), while satisfying the equality in the Heisenberg uncertainty principle $\Delta {X}_{\mathrm{min}}\Delta {X}_{\mathrm{max}}=1/4$ [red dashed line in Panels c and g] with the variance $(\Delta {X}_{\mathrm{max}}{)}^{2}$ [red bold dash-dotted line in Panels c and g] conjugate to $(\Delta {X}_{\mathrm{min}}{)}^{2}$.

These are plotted as a function of $g/{\omega }_{a}$ in Fig. 5a, e with ${\omega }_{b}={\omega }_{a}$ and ${\omega }_{b}=2{\omega }_{a}$; the latter was chosen as an example of the detuned cases. The zero-temperature SRPT occurs at

6 $$g = \frac{{\sqrt {\omega_{a} \omega_{b} } }}{2}.$$

In this way, it occurs in the ultrastrong or deep strong coupling regime^20–22^.

The quantum fluctuations around the zero-temperature classical state are described by replacing $\widehat{a}$ and $\widehat{b}$ with $\overline{a }\sqrt{N}+\widehat{a}$ and $-\overline{b }\sqrt{N}+\widehat{b}$, respectively^14,15,40,41^. Thereafter, $\widehat{a}$ and $\widehat{b}$ are considered as fluctuation operators. The Dicke Hamiltonian, Eq. (1), is expanded as

7 $$\widehat{\mathcal{H}}/\hslash \equiv N\overline{\mathcal{H} }/\mathrm{\hslash }+{\omega }_{a}{\widehat{a}}^{\dagger}\widehat{a}+{\widetilde{\omega }}_{b}{\widehat{b}}^{\dagger}\widehat{b}+\widetilde{g}({\widehat{a}}^{\dagger}+\widehat{a})({\widehat{b}}^{\dagger}+\widehat{b})+\widetilde{D}({\widehat{b}}^{\dagger}+\widehat{b}{)}^{2}+O({N}^{-1/2}),$$

where the coefficients are modified by the order parameters $\overline{a }$ and $\overline{b }$:

8 $$\tilde{g} \equiv \frac{{g\left( {1 - 2\overline{b}^{2} } \right)}}{{\sqrt {1 - \overline{b}^{2} } }},\;\;\tilde{D} \equiv \frac{{g\overline{a}\overline{b}}}{{\sqrt {1 - \overline{b}^{2} } }},\;\;\tilde{\omega }_{b} \equiv \omega_{b} + 2\tilde{D}.$$

In Eq. (7), the first term represents the classical energy $N\overline{\mathcal{H} }$ governing the zero-temperature SRPT. The quadratic Hamiltonian in terms of $\widehat{a}$ and $\widehat{b}$ represent the energy of quantum fluctuation from the zero-temperature classical state. The higher order terms are of the order of ${N}^{-1/2}$. By numerically diagonalizing the original Dicke model with increasing the number of atoms (as we have performed in previous subsection), it has been confirmed that the lowest transition frequencies^9^, quantum entanglement and pairwise concurrence^16^ asymptotically approach those obtained by the quadratic Hamiltonian in the thermodynamic limit (neglecting the higher order terms). In the following, we focus only on the quadratic Hamiltonian for discussing the quantum squeezing in the thermodynamic limit ($N\to \infty$). The calculation will be similar to that for the quantum squeezing generation by the optical parametric oscillation^31,32^. However, optical parametric oscillation is a primarily non-equilibrium spontaneous symmetry breaking (critical) phenomenon, whereas the SRPT in the present study occurs in thermal equilibrium.

By describing the photonic and atomic fluctuations using Eq. (7), we will demonstrate numerically the perfect intrinsic two-mode squeezing. We consider a general superposition (two-mode basis^31,32^) of the two fluctuation operators defined in terms of two angles $\theta$ and $\psi$:

9 $${\widehat{c}}_{\theta ,\psi }\equiv \widehat{a}\mathrm{cos}\theta +{\mathrm{e}}^{\mathrm{i}\psi }\widehat{b}\mathrm{sin}\theta .$$

In the case of $\psi =0$, $\theta$ corresponds to the angle depicted in Figs. 2 and 4. We define a quadrature^31,32^ with the bosonic operator ${\widehat{c}}_{\theta ,\psi }$ and phase $\varphi$ as

10 $${\widehat{X}}_{\theta ,\psi ,\varphi }=({\widehat{c}}_{\theta ,\psi }{\mathrm{e}}^{\mathrm{i}\varphi }+{\widehat{c}}_{\theta ,\psi }^{\dagger}{\mathrm{e}}^{-\mathrm{i}\varphi })/2.$$

In the case of $\theta =-\pi /4$ and $\psi =0$, ${\widehat{X}}_{-\pi /\mathrm{4,0},0}$ ($\varphi =0$) and ${\widehat{X}}_{-\pi /\mathrm{4,0},\pi /2}$ ($\varphi =\pi /2$) correspond to the operators of $\left({\alpha }_{\mathrm{r}}-{\beta }_{\mathrm{r}}\right)/\sqrt{2}$ and $\left({\alpha }_{\mathrm{i}}-{\beta }_{\mathrm{i}}\right)/\sqrt{2}$, respectively, discussed in the previous subsection. We evaluate the variance $(\Delta {X}_{\theta ,\psi ,\varphi }{)}^{2}\equiv \langle 0|({\widehat{X}}_{\theta ,\psi ,\varphi }{)}^{2}|0\rangle -\langle 0|{\widehat{X}}_{\theta ,\psi ,\varphi }|0{\rangle }^{2}=\langle 0|({\widehat{X}}_{\theta ,\psi ,\varphi }{)}^{2}|0\rangle$ of this quadrature with respect to the ground state $|0\rangle$ of the fluctuation Hamiltonian, Eq. (7).

Here, we consider annihilation operators ${\widehat{p}}_{\pm }$ of eigenmodes (i.e., polariton modes) that diagonalize Eq. (7) as

11 $$\widehat{\mathcal{H}}/\hslash ={\Omega }_{-}{\widehat{p}}_{-}^{\dagger}{\widehat{p}}_{-}+{\Omega }_{+}{\widehat{p}}_{+}^{\dagger}{\widehat{p}}_{+}+O({N}^{-1/2})+\mathrm{const}.,$$

where ${\Omega }_{\pm }$ are the eigenfrequencies. The ground state $|0\rangle$ is defined such that

12 $${\widehat{p}}_{\pm }|0\rangle =0.$$

Owing to the presence of the counter-rotating terms $\widehat{a}\widehat{b}$, ${\widehat{a}}^{\dagger}{\widehat{b}}^{\dagger}$, $\widehat{b}\widehat{b}$, and ${\widehat{b}}^{\dagger}{\widehat{b}}^{\dagger}$, originating from those in the Dicke model in Eq. (1), the eigenmode operators are obtained via Bogoliubov transformation^20,23–27,40,41^:

13 $${\widehat{p}}_{\pm }={w}_{\pm }\widehat{a}+{x}_{\pm }\widehat{b}+{y}_{\pm }{\widehat{a}}^{\dagger}+{z}_{\pm }{\widehat{b}}^{\dagger}.$$

For positive eigenfrequencies ${\Omega }_{\pm }>0$ (when energy is needed to excite the eigenmodes), the coefficients must satisfy $|{w}_{\pm }{|}^{2}+|{x}_{\pm }{|}^{2}-|{y}_{\pm }{|}^{2}-|{z}_{\pm }{|}^{2}=1$ to yield $[{\widehat{p}}_{\pm },{\widehat{p}}_{\pm }^{\dagger}]=1$. These coefficients and ${\Omega }_{\pm }$ are determined by an eigenvalue problem^20,41^ derived from Eq. (7):

14 $$\left(\begin{array}{llll}{\omega }_{a}& \widetilde{g}& 0& -\widetilde{g}\\ \widetilde{g}& {\widetilde{\omega }}_{b}+2\widetilde{D}& -\widetilde{g}& -2\widetilde{D}\\ 0& \widetilde{g}& -{\omega }_{a}& -\widetilde{g}\\ \widetilde{g}& 2\widetilde{D}& -\widetilde{g}& -{\widetilde{\omega }}_{b}-2\widetilde{D}\end{array}\right)\left(\begin{array}{l}{w}_{\pm }\\ {x}_{\pm }\\ {y}_{\pm }\\ {z}_{\pm }\end{array}\right)={\Omega }_{\pm }\left(\begin{array}{l}{w}_{\pm }\\ {x}_{\pm }\\ {y}_{\pm }\\ {z}_{\pm }\end{array}\right).$$

Two positive eigenvalues correspond to the eigenfrequencies ${\Omega }_{\pm }$. We also obtain two negative eigenvalues $-{\Omega }_{\pm }$, which are mathematically obtained by solving Eq. (14), and their eigenvectors correspond to the creation operators ${\widehat{p}}_{\pm }^{\dagger}$. In this study, we suppose $0\le {\Omega }_{-}\le {\Omega }_{+}$; that is, ${\Omega }_{-}$ and ${\Omega }_{+}$ are the eigenfrequencies of the lower and upper eigenmodes, respectively. Figure 5b, f shows ${\Omega }_{\pm }$ as functions of $g/{\omega }_{a}$. It is known that the lower eigenfrequency ${\Omega }_{-}$ vanishes at the SRPT critical point $g=\sqrt{{\omega }_{a}{\omega }_{b}}/2$^14,15^. In this case, $[{\widehat{p}}_{-},{\widehat{p}}_{-}^{\dagger}]=1$ does not hold because Eq. (14) yields two mathematically degenerate solutions with ${\Omega }_{-}=0$. In the following, we will see that perfect squeezing is obtained at this critical point.

The quadrature variance $(\Delta {X}_{\theta ,\psi ,\varphi }{)}^{2}=\langle 0|({\widehat{X}}_{\theta ,\psi ,\varphi }{)}^{2}|0\rangle$ can be evaluated by rewriting the original photonic and atomic fluctuation operators $\widehat{a}$, ${\widehat{a}}^{\dagger}$, $\widehat{b}$, and ${\widehat{b}}^{\dagger}$ in terms of the eigenmode operators ${\widehat{p}}_{\pm }$ and ${\widehat{p}}_{\pm }^{\dagger}$ and using Eq. (12). We numerically searched for the optimal angles ${\theta }_{\mathrm{opt}}$, ${\psi }_{\mathrm{opt}}$, and ${\varphi }_{\mathrm{opt}}$ that provide the minimum variance $(\Delta {X}_{\mathrm{min}}{)}^{2}\equiv (\Delta {X}_{{\theta }_{\mathrm{opt}},{\psi }_{\mathrm{opt}},{\varphi }_{\mathrm{opt}}}{)}^{2}$ for given ${\omega }_{a}$, ${\omega }_{b}$, and $g$.

In Fig. 5c, g, quadrature variances, including $(\Delta {X}_{\mathrm{min}}{)}^{2}$, and (d,h) optimal angles ${\theta }_{\mathrm{opt}}$, ${\psi }_{\mathrm{opt}}$, and ${\varphi }_{\mathrm{opt}}$ are plotted as functions of $g/{\omega }_{a}$ for (c,d) ${\omega }_{b}={\omega }_{a}$ and (g,h) ${\omega }_{b}=2{\omega }_{a}$. As shown by the red bold solid lines in Fig. 5c, g, while the minimum variance is $(\Delta {X}_{\mathrm{min}}{)}^{2}=1/4$ (standard quantum limit^31,32^) in the absence of photon–atom coupling ($g=0$), it decreases as $g$ increases and vanishes (perfect squeezing is obtained) at the SRPT critical point, $g=\sqrt{{\omega }_{a}{\omega }_{b}}/2$. Subsequently, in the superradiant phase ($g>\sqrt{{\omega }_{a}{\omega }_{b}}/2$), $(\Delta {X}_{\mathrm{min}}{)}^{2}$ increases again and approaches $1/4$ asymptotically.

In this way, by using the quadratic Hamiltonian in the thermodynamic limit (neglecting the higher order terms), we get the perfect squeezing $(\Delta {X}_{\mathrm{min}}{)}^{2}\to 0$ at the SRPT critical point. Its validity can be confirmed from the asymptotic behavior (monotonic decrease) of $(\Delta {X}_{\mathrm{min}}{)}^{2}$ seen in Fig. 4g in the case of ${\omega }_{b}={\omega }_{a}$ (we have confirmed also for detuned cases, while not shown in figures).

Next, we calculated the variance $(\Delta {X}_{\mathrm{max}}{)}^{2}\equiv (\Delta {X}_{{\theta }_{\mathrm{opt}},{\psi }_{\mathrm{opt}},{\varphi }_{\mathrm{opt}}-\pi /2}{)}^{2}$ of the quadrature ${X}_{{\theta }_{\mathrm{opt}},{\psi }_{\mathrm{opt}},{\varphi }_{\mathrm{opt}}-\pi /2}$ conjugate to the optimal one ${X}_{{\theta }_{\mathrm{opt}},{\psi }_{\mathrm{opt}},{\varphi }_{\mathrm{opt}}}$; $(\Delta {X}_{\mathrm{max}}{)}^{2}$ is represented by the red bold dash-dotted lines in Fig. 5c, g. We found that this variance diverges at the SRPT critical point. However, as shown by the red bold dashed lines in Fig. 5c, g, we numerically confirmed that the product satisfies $\Delta {X}_{\mathrm{min}}\Delta {X}_{\mathrm{max}}=1/4$, although only an inequality $\Delta {X}_{\mathrm{min}}\Delta {X}_{\mathrm{max}}\ge 1/4$ is obtained in general.

In this way, in the thermodynamic limit and under the Holstein–Primakoff transformation, the quantum fluctuation in the ground state $|0\rangle$ is not simply squeezed but also satisfies the equality in the Heisenberg uncertainty principle (i.e., ideal two-mode squeezing is obtained). However, as we have seen in Fig. 4h, we did not confirm that $\Delta {X}_{\mathrm{min}}\Delta {X}_{\mathrm{max}}$ asymptotically reaches $1/4$ correctly with the increase in $N$. This confirmation remains as a future task.

In Fig. 5c, g, the blue thin solid lines represent the variance $(\Delta {X}_{\mathrm{0,0},\pi /2}{)}^{2}=\langle 0|(\widehat{a}-{\widehat{a}}^{\dagger}{)}^{2}|0\rangle /4$ of a photonic fluctuation. As we have already discussed in the previous subsection, this type of one-mode variance does not vanish even at the critical point^15,17,19^. Further, as shown by the thin blue dashed line, the one-mode squeezing satisfies only the inequality $\Delta {X}_{\mathrm{0,0},\pi /2}\Delta {X}_{\mathrm{0,0},0}>1/4$ in the Heisenberg uncertainty principle even in the thermodynamic limit and under the Holstein–Primakoff transformation.

As seen in Fig. 5d, h, in the present case, the minimum variance is always obtained for ${\psi }_{\mathrm{opt}}=0$ (dashed line) and ${\varphi }_{\mathrm{opt}}=\pi /2$ (dash-dotted line). These two phases depend on those of the coupling strengths of the co- and counter-rotating terms^42^, although we simply considered the isotropic Dicke model, Eq. (1), and real $g$ in the present calculation. Conversely, ${\theta }_{\mathrm{opt}}$ (solid curves) depends on $g/{\omega }_{a}$ and ${\omega }_{b}/{\omega }_{a}$ in general, while ${\theta }_{\mathrm{opt}}=-\pi /4$; that is, $(\Delta {X}_{-\pi /\mathrm{4,0},\pi /2}{)}^{2}$, Eq. (2), always yields the minimum variance in the normal phase ($g<{\omega }_{a}/2)$ for ${\omega }_{b}={\omega }_{a}$. It is consistent with what we found in Figs. 2, 3, and 4.

## Discussion

We numerically found that the minimum variance vanishes $[{\left(\Delta {X}_{\mathrm{min}}\right)}^{2}\to 0$] and its conjugate variance diverges [${\left(\Delta {X}_{\mathrm{max}}\right)}^{2}\to \infty$], i.e., squeezing becomes *perfect* at the SRPT critical point in the thermodynamic limit ($N\to \infty$). This occurs when we choose an appropriate photon–atom two-mode basis, as in Fig. 5c, g. Here, ${\left(\Delta {X}_{\mathrm{min}}\right)}^{2}$ and ${\left(\Delta {X}_{\mathrm{max}}\right)}^{2}$ were calculated from the fluctuation Hamiltonian, Eq. (7), derived from the Dicke model, Eq. (1), through the Holstein–Primakoff transformation and by considering the spontaneous symmetry breaking in the thermodynamic limit. The asymptotic behavior to the perfect squeezing [${\left(\Delta {X}_{\mathrm{min}}\right)}^{2}\to 0$] was confirmed in Fig. 4g by increasing $N$.

As pointed out by Hirsch et al*.*^43^, such Hamiltonians derived by truncating the terms [$O({N}^{-1/2})$ in Eq. (7)] beyond the quadratic ones may show some divergent (singular) results that are not obtained in the original Hamiltonians. However, as demonstrated by Emary and Brandes^15^ and also in Figs. 2 and 4 of the present study, the signature of divergence (anti-squeezing) along the axis of $\left({\alpha }_{\mathrm{r}}-{\beta }_{\mathrm{r}}\right)/\sqrt{2}$ was observed by numerically diagonalization of the original Dicke model for finite $N$. It indicates that the divergent ${\left(\Delta {X}_{\mathrm{max}}\right)}^{2}$ and vanishing ${\left(\Delta {X}_{\mathrm{min}}\right)}^{2}$ in the thermodynamic limit are not an artifact caused by the truncation of the higher-order terms.

Note also that, although the numerical calculation cannot be performed at exactly the critical point ($g=\sqrt{{\omega }_{a}{\omega }_{b}}/2$) due to the divergence for the fluctuation Hamiltonian, Eq. (7), it is numerically observed that the minimum variance gradually vanishes [${\left(\Delta {X}_{\mathrm{min}}\right)}^{2}\to 0$] when the coupling strength approaches the critical value ($g\to \sqrt{{\omega }_{a}{\omega }_{b}}/2+{0}^{\pm }$). This also indicates that the truncated Hamiltonian is justified even near the critical point as long as the number $N$ of atoms is larger than the expectation number $\left.\langle 0\right|{\widehat{b}}^{\dagger}\widehat{b}\left|0\right.\rangle$ of atomic excitations, which becomes infinite only at exactly the critical point.

A detailed mathematical analysis of the perfect squeezing [${\left(\Delta {X}_{\mathrm{min}}\right)}^{2}\to 0$] is presented in “Methods”, where we derived analytical expressions of the ground state $|0\rangle$ of the fluctuation Hamiltonian, Eq. (7). In the case of ${\omega }_{b}={\omega }_{a}$ and the normal phase ($g<\sqrt{{\omega }_{a}{\omega }_{b}}/2$), we could easily find that the ground state is expressed as $|0\rangle \propto {\widehat{U}}_{d-}{\widehat{U}}_{d+}|{0}_{a,b}\rangle$, where ${\widehat{d}}_{\pm }=(\widehat{a}\pm \widehat{b})/\sqrt{2}$ are the equal-weight superpositions of the original fluctuation operators, ${\widehat{U}}_{d\pm }={\mathrm{e}}^{(-{r}_{\pm }/2)({\widehat{d}}_{\pm }^{\dagger}{\widehat{d}}_{\pm }^{\dagger}-{\widehat{d}}_{\pm }{\widehat{d}}_{\pm })}$ are squeezing operators in that basis, and $\left|{0}_{a,b}\right.\rangle \equiv {\left|n\right.\rangle }_{a}{\left|\frac{N}{2},-\frac{N}{2}\right.\rangle }_{S}$ is the original vacuum satisfying $\widehat{a}|{0}_{a,b}\rangle =\widehat{b}|{0}_{a,b}\rangle =0$. This is analytical evidence indicating why $\Delta {X}_{\mathrm{min}}\Delta {X}_{\mathrm{max}}=1/4$ is satisfied for any $g$ at ${\theta }_{\mathrm{opt}}=-\pi /4$, ${\psi }_{\mathrm{opt}}=0$, and ${\varphi }_{\mathrm{opt}}=\pi /2$ in the fluctuation Hamiltonian, Eq. (7), because $|0\rangle$ is an ideal two-mode squeezed vacuum where the variances of quadratures defined by ${\widehat{d}}_{-}={\widehat{c}}_{-\pi /\mathrm{4,0}}=(\widehat{a}-\widehat{b})/\sqrt{2}$ obey $(\Delta {X}_{\mathrm{min}}{)}^{2}=(\Delta {X}_{-\pi /\mathrm{4,0},\pi /2}{)}^{2}={\mathrm{e}}^{2{r}_{-}}/4$ and $(\Delta {X}_{\mathrm{max}}{)}^{2}=(\Delta {X}_{-\pi /\mathrm{4,0},0}{)}^{2}={\mathrm{e}}^{-2{r}_{-}}/4$. From the analytical expression of ${r}_{-}$ in Eq. (33), we could also easily find that the perfect squeezing is obtained as ${r}_{-}\to -\infty$ in the ${\widehat{d}}_{-}$ basis when the coupling strength reaches the critical point as $g\to {\omega }_{a}/2+{0}^{-}$. This is analytical evidence indicating why the quadrature variance $(\Delta {X}_{\mathrm{min}}{)}^{2}$ vanishes at the SRPT critical point, as demonstrated in Fig. 5. From the expression of the ground state derived in “Methods”, we can mathematically confirm perfect squeezing also in the general case with ${\omega }_{b}\ne {\omega }_{a}$ (and in the superradiant phase). Instead of such a straightforward but complicated analysis, we can also understand perfect squeezing at the SRPT critical point $g=\sqrt{{\omega }_{a}{\omega }_{b}}/2$ in the following manner.

Perfect squeezing can generally be obtained when the quadrature ${\widehat{X}}_{\theta ,\psi ,\varphi }=[({\mathrm{e}}^{\mathrm{i}\varphi }\widehat{a}+{\mathrm{e}}^{-\mathrm{i}\varphi }{\widehat{a}}^{\dagger})\mathrm{cos}\theta +{\mathrm{e}}^{\mathrm{i}\psi }({\mathrm{e}}^{\mathrm{i}\varphi }\widehat{b}+{\mathrm{e}}^{-\mathrm{i}\varphi }{\widehat{b}}^{\dagger})\mathrm{sin}\theta ])/2$ is proportional to the eigenmode operator ${\widehat{p}}_{-}$, because ${\widehat{p}}_{-}|0\rangle =0$ and then the quadrature variance $\langle 0|({\widehat{X}}_{\theta ,\psi ,\varphi }{)}^{2}|0\rangle$ becomes zero. As we can freely choose the angles $\theta$, $\psi$, and $\varphi$, perfect squeezing can be obtained when the weights of the annihilation and creation operators in the eigenmode operator ${\widehat{p}}_{-}={w}_{-}\widehat{a}+{x}_{-}\widehat{b}+{y}_{-}{\widehat{a}}^{\dagger}+{z}_{-}{\widehat{b}}^{\dagger}$ are equal as $|{w}_{-}|=|{y}_{-}|$ and $|{x}_{-}|=|{z}_{-}|$. Such equal weights are obtained at critical points accompanied by a vanishing resonance frequency in some interacting systems (e.g., weakly interacting Bose gases^44^). In the present case, we can easily find that ${w}_{-}/{y}_{-}={x}_{-}/{z}_{-}=-1$ is obtained under the condition of ${\Omega }_{-}=0$ from the eigenvalue problem in Eq. (14). Thus, we can generally obtain perfect squeezing in an appropriate quadrature at critical points in the Dicke model, as well as in similar models with counter-rotating terms and a vanishing resonance frequency.

In summary, we found that perfect squeezing is an intrinsic property associated with the zero-temperature SRPT in the Dicke model in the thermodynamic limit ($N\to \infty$). Phenomenologically, owing to a possible divergence of quantum fluctuation [e.g., along $\left({\alpha }_{\mathrm{r}}-{\beta }_{\mathrm{r}}\right)/\sqrt{2}$ axis in Fig. 4a, c, e demonstrated for finite $N$] at a critical point, its conjugate fluctuation can be perfectly squeezed [e.g., along $\left({\alpha }_{\mathrm{i}}-{\beta }_{\mathrm{i}}\right)/\sqrt{2}$ axis in Fig. 4b, d, f demonstrated for finite $N$] while satisfying the Heisenberg uncertainty principle. Such a quantum behavior should be obtained only in limited systems with a vanishing resonance frequency and counter-rotating terms; we confirmed that the Dicke model is one of such systems.

In the superradiant phase, the physical quantities that mediate the photon–atom coupling get non-zero values spontaneously, and their conjugate variables are perfectly squeezed (two quantum fluctuations are perfectly synchronized) at the SRPT critical point. For instance, if the photon–atom coupling is mediated by the electric (displacement) field $D$ and electric polarization $P$, non-zero $D$ and $P$ appear spontaneously in the superradiant phase, and the quantum fluctuations of the vector potential $A$ and electric current $J$ are perfectly synchronized (squeezed) at the SRPT critical point.

By the standard squeezing generation processes in dynamic and nonequilibrium situations, the two-mode squeezed vacuum ${\widehat{U}}_{d-}{\widehat{U}}_{d+}|{0}_{a,b}\rangle$ is also generated and perfect squeezing (${r}_{\pm }\to -\infty$) can be obtained at dynamical critical points such as at the threshold of the optical parametric oscillation^31,32^. However, this perfect squeezed vacuum is an *excited* state of the photonic system in free space (flying photons) carrying an infinite energy (infinite number of photons), whose Hamiltonian is given by ${\widehat{\mathcal{H}}}_{\mathrm{free}}/\hslash \equiv {\omega }_{a}{\widehat{a}}^{\dagger}\widehat{a}+{\widetilde{\omega }}_{b}{\widehat{b}}^{\dagger}\widehat{b}$. By contrast, in the Dicke model, the squeezed vacuum and perfect squeezing are obtained in the *ground* state, i.e., in the energetically minimal state. Although photon loss (dissipation) can generate quantum entanglement and squeezing in some specially designed driven-dissipative situations^45,46^, usually squeezing of flying photons easily diminishes due to photon loss during generation, propagation, and detection and due to noise in the driving laser light, nonlinear crystal, cavity mirrors, etc.^47^. In contrast, the phenomenon of intrinsic squeezing described here does not diminish with time and is stably obtained in equilibrium situations.

Therefore, intrinsic squeezing might have the potential to make quantum sensing^33^ and continuous-variable quantum information technologies^34,35^ intrinsically robust against the photon loss and noises (decoherence). We can use some of the existing protocols by replacing the superposing and displacement operations for flying photons with those for photons in equilibrium, which can be implemented via adiabatic changes in system parameters. The control of system parameters is well established in superconducting circuits and also in magnonic systems^42^, both of which can show the equilibrium SRPTs^9,10^. Specifically, ongoing terahertz magnetospectroscopy measurements of Er_*x*_Y_1-*x*_FeO_3_^48^ provide us an experimental platform for creating squeezed magnons around the magnonic SRPT in thermal equilibrium, whereas we need a different technique for measuring the intrinsic squeezing.

Concerning the measurements, in contrast to the perfect intrinsic spin squeezing reported in some spin models, such as the Lipkin–Meshkov–Glick model^28^, the XY model^29^, and the transverse-field Ising model^30^, the quantum fluctuations of photons can be measured using modern experimental techniques even in the ground state and in general equilibrium situations^49,50^. The quantum fluctuations of magnons can also be performed in a similar manner by utilizing a magnonic nonlinearity.

Although we restricted the present investigation only to zero temperature for deriving simple analytical expressions, the obtained intrinsic squeezing is expected to become imperfect at finite temperatures. Such investigations should be performed for practical applications, including quantum metrology^30^, for example, along the calculation scheme of Shapiro, Pogosov, and Lozovik^19^. The asymptotic behavior of $\Delta {X}_{\mathrm{min}}\Delta {X}_{\mathrm{max}}$ with the increase in $N$ should also be analyzed more in detail. Further, whereas we implicitly assumed that system–bath coupling is much weaker than the system parameters (${\omega }_{a,b}$ and $g$), it should also worsen the intrinsic squeezing. Such an influence should be investigated, for example, using the scheme by Shitara et al*.*^51^. Although we considered the isotropic Dicke model in the present study for deriving simple analytical expressions, we confirmed numerically that perfect intrinsic squeezing can be obtained even within the anisotropic Dicke model, where the co- and counter-rotating coupling strengths are different^40^ and complex. By multiplying phase factors to the photonic and atomic operators as $\widehat{a}\to {\mathrm{e}}^{-\mathrm{i}{\phi }_{a}}\widehat{a}$ and ${\widehat{S}}_{-}\to {\mathrm{e}}^{-\mathrm{i}{\phi }_{b}}{\widehat{S}}_{-}$, the coupling term is transformed to $({g}_{1}{\widehat{a}}^{\dagger}{\widehat{S}}_{-}+{g}_{1}^{*}{\widehat{S}}_{+}\widehat{a})/\sqrt{N}+({g}_{2}{\widehat{a}}^{\dagger}{\widehat{S}}_{+}+{g}_{2}^{*}{\widehat{S}}_{-}\widehat{a})/\sqrt{N}$, and we obtain complex coupling strengths ${g}_{1}=g{\mathrm{e}}^{\mathrm{i}({\phi }_{a}-{\phi }_{b})}$ and ${g}_{2}=g{\mathrm{e}}^{\mathrm{i}({\phi }_{a}+{\phi }_{b})}$ for the co- and counter-rotating terms, respectively. Thus, our analytical results can be applied for solving cases with complex coupling strengths. We can numerically confirm that the optimal phases are ${\psi }_{\mathrm{opt}}={\phi }_{a}-{\phi }_{b}$ and ${\varphi }_{\mathrm{opt}}=\pi /2-{\phi }_{a}$ for the complex coupling strengths. However, it is still an open question whether perfect squeezing can be obtained in more realistic systems beyond the Dicke model. Such studies are required for examining sensing and computing protocols in superconducting circuits^9^ and ErFeO_3_^10^ that show SRPTs in equilibrium.

## Methods

### Numerical evaluation of Wigner functions and variances

For calculating the ground-state wavefunction of the Dicke model by the Wigner function, we first define the atomic Fock state with the total angular momentum of $\hslash S=\hslash N/2$ as

15 $$\left| n \right. \rangle_{b} \equiv \left| {\frac{N}{2}, n - \frac{N}{2}} \right. \Bigg\rangle_{S} ,$$

where $n = 0, 1, 2, \ldots , N$. We define the atomic coherent state as

16 $$\left| \beta \right. \rangle_{b} \equiv {\text{e}}^{{ - \frac{{\left| \beta \right|^{2} }}{2}}} \mathop \sum \limits_{n = 0}^{N} \frac{{\beta^{n} }}{{\sqrt {n!} }}\left| n \right. \rangle_{b} .$$

Thus, the two-mode coherent state with a photonic complex amplitude $\alpha \in {\mathbb{C}}$ and an atomic one $\beta \in {\mathbb{C}}$ is defined as

17 $$\left| {\alpha ,\beta } \right. \rangle \equiv {\text{e}}^{{\alpha \hat{a}^{\dagger } - \alpha^{*} \hat{a}}} \left| 0 \right. \rangle _{a} \left| \beta \right. \rangle_{b} .$$

Because the ground state $\left| 0 \right\rangle$ is a pure state, the Q function^31^ is represented as

18 $$Q_{ab} \left( {\alpha ,\beta } \right) \equiv \frac{{\left| \langle {\alpha , \beta |0}\rangle \right|^{2} }}{{\pi^{2} }}.$$

Here, we rewrite the Q function by introducing diagonal variable $\xi_{ \pm } \equiv \left( {\alpha \pm \beta } \right)/\sqrt 2$ as

19 $$Q_{c} \left( {\xi_{ + } , \xi_{ - } } \right) \equiv Q_{ab} \left( {\frac{{\xi_{ + } + \xi_{ - } }}{\sqrt 2 },\frac{{\xi_{ + } - \xi_{ - } }}{\sqrt 2 }} \right).$$

Because of the limitation of our computational power, we transform this Q function into the Wigner function^31^ only along the $\xi_{ - }$ direction, which corresponds to the axis of the squeezing and anti-squeezing as seen in Figs. 2 and 4. We define the anti-normally ordered characteristic function as

20 $$C_{A} \left( {\xi_{ + } ,\lambda } \right) \equiv \int {\text{d}}^{2} \xi_{ - }\ Q_{c} \left( {\xi_{ + } ,\xi_{ - } } \right){\text{e}}^{{\lambda \xi_{ - }^{*} - \lambda^{*} \xi_{ - } }} .$$

The symmetrically ordered characteristic function is calculated as

21 $$C_{S} \left( {\xi_{ + } ,\lambda } \right) \equiv C_{A} \left( {\xi_{ + } ,\lambda } \right){\text{e}}^{{\left| \lambda \right|^{2} /2}} .$$

Using this, we calculate the Wigner function as

22 $$W\left( {\xi_{ + } ,\xi_{ - } } \right) \equiv \frac{{{\text{e}}^{{ - \left| {\xi_{ + } } \right|^{2} }} }}{{\pi^{2} }}\int {\text{d}}^{2} \lambda\ C_{S} \left( {\xi_{ + } ,\lambda } \right){\text{e}}^{{\xi_{ - } \lambda^{*} - \xi_{ - }^{*} \lambda }} .$$

Here, the factor $e^{{ - \left| {\xi_{ + } } \right|^{2} }}$ is additionally multiplied for compensating the broadening difference between the Wigner function (along $\xi_{ - }$ axis) and Q function (along $\xi_{ + }$ axis).

For numerically evaluating the variances in Eqs. (2) and (3), using the atomic Fock states in Eq. (15), we define the atomic annihilation operator as

23 $$\hat{b} \equiv \mathop \sum \limits_{n = 0}^{N} \sqrt {n + 1} \left| n \right\rangle_{bb} \left\langle {n + 1} \right|.$$

In the numerical calculations, the operators including the Hamiltonian are represented as matrices on the basis of the two-mode Fock states ${\left.|n\right\rangle }_{a}{\left.|{n}^{\prime}\right\rangle }_{b}={\left.|n\right\rangle }_{a}{\|\frac{N}{2}, {n}^{\prime}-\frac{N}{2} \left.\right\rangle }_{S}$, whereas the states with $n>{N}_{\mathrm{max}}$ or $n+{n}^{^{\prime}}>{N}_{\mathrm{max}}^{^{\prime}}$ are truncated. We have numerically confirmed that the results in Figs. 2, 3, and 4 are well saturated by using large enough ${N}_{\mathrm{max}}$ and ${N}_{\mathrm{max}}^{^{\prime}}$.

We set ${N}_{\mathrm{max}}=112$ and ${N}_{\mathrm{max}}^{^{\prime}}=114$ for Figs. 2a–f and 4a–d. ${N}_{\mathrm{max}}=146$ and ${N}_{\mathrm{max}}^{^{\prime}}=148$ for Figs. 2g–j, 4e, f. For Fig. 3, we set ${N}_{\mathrm{max}}^{^{\prime}}={N}_{\mathrm{max}}+N$. ${N}_{\mathrm{max}}=200$ for Fig. 3a, c, and e, red dashed and blue dash-dotted lines in Fig. 3b, d, f. ${N}_{\mathrm{max}}=1500$ for yellow lines in Fig. 3b, d, f.

For Fig. 4g, h, we set ${N}_{\mathrm{max}}=10000$ and ${N}_{\mathrm{max}}^{^{\prime}}=10002$. We have confirmed that the additional truncation (${N}_{\mathrm{max}}^{^{\prime}}={N}_{\mathrm{max}}+2$) does not change the calculated ${\left(\Delta {X}_{\mathrm{min}}\right)}^{2}$ and $\Delta {X}_{\mathrm{min}}\Delta {X}_{\mathrm{max}}$ by comparing those without the truncation (${N}_{\mathrm{max}}^{^{\prime}}={N}_{\mathrm{max}}+N$) up to $N={2}^{18}\approx 2.6\times {10}^{5}$ with ${N}_{\mathrm{max}}=1000.$

### Analytical expression of the squeezed ground state

Here, we explain the numerically found perfect and ideal squeezing ($\Delta {X}_{\mathrm{min}}=0$ at the critical point with $\Delta {X}_{\mathrm{min}}\Delta {X}_{\mathrm{max}}=1/4$) using an analytical expression of the ground state $|0\rangle$ of the fluctuation Hamiltonian, i.e., Eq. (7). Following the discussion by Schwendimann and Quattropani^25–27^, we consider a unitary operator $\widehat{U}$ that transforms the fluctuation operators $\widehat{a}$ and $\widehat{b}$ into the eigenmode operators ${\widehat{p}}_{\pm }$ as

24 $$\hat{p}_{ - } \equiv \hat{U}\hat{a}\hat{U}^{\dagger } ,\;\hat{p}_{ + } \equiv \hat{U}\hat{b}\hat{U}^{\dagger } .$$

For the vacuum $\left|{0}_{a,b}\right.\rangle ={\left|0\right.\rangle }_{a}{\left|0\right.\rangle }_{b}$ of the individual fluctuations satisfying $\widehat{a}|{0}_{a,b}\rangle =\widehat{b}|{0}_{a,b}\rangle =0$, the ground state $|0\rangle$ of the coupled system can be expressed as

25 $$\left| {0\rangle \propto \hat{U}} \right|0_{a,b} \rangle ,$$

while there exists freedom to introduce an overall phase factor. This expression satisfies Eq. (12).

Sharma and Kumar^40^ recently showed the explicit expression of $\widehat{U}$ for the fluctuation Hamiltonian, Eq. (7), derived from the Dicke model, as

26 $$\hat{U} \equiv \hat{U}_{0} \hat{U}_{ - } \hat{U}_{ + } ,$$

where the three unitary operators are defined as

27 $$\hat{U}_{0} \equiv {\text{e}}^{{ - \left( {r_{b} /2} \right)\left( {\hat{b}^{\dagger } \hat{b}^{\dagger } - \hat{b}\hat{b}} \right)}} {\text{e}}^{{ - \phi \left( {\hat{a}^{\dagger } \hat{b} - \hat{b}^{\dagger } \hat{a}} \right)}} {\text{e}}^{{ - r\left( {\hat{a}^{\dagger } \hat{b}^{\dagger } - \hat{b}\hat{a}} \right)}} ,$$

28 $$\hat{U}_{ - } \equiv {\text{e}}^{{ - \left( {r_{ - } /2} \right)\left( {\hat{a}^{\dagger } \hat{a}^{\dagger } - \hat{a}\hat{a}} \right)}} ,\;\hat{U}_{ + } \equiv {\text{e}}^{{ - \left( {r_{ + } /2} \right)\left( {\hat{b}^{\dagger } \hat{b}^{\dagger } - \hat{b}\hat{b}} \right)}} .$$

Here, ${\widehat{U}}_{\pm }$ are one-mode squeezing operators, and ${\widehat{U}}_{0}$ is a product of one-mode squeezing, superposing, and two-mode squeezing operators^31,32^. Using a Bogoliubov transformation of $\widehat{b}$ for renormalizing the $\widetilde{D}$ term in Eq. (7), the atomic frequency and coupling strength are modified as follows:

29 $$\overset{\lower0.5em\hbox{$\smash{\scriptscriptstyle\smile}$}}{\omega }_{b} \equiv \sqrt {\tilde{\omega }_{b} \left( {\tilde{\omega }_{b} + 4\tilde{D}} \right)} ,\;\;\overset{\lower0.5em\hbox{$\smash{\scriptscriptstyle\smile}$}}{g} \equiv \sqrt {\left( {1 - \gamma } \right)/\left( {1 + \gamma } \right)} \tilde{g}$$

where $\gamma$, also yielding $r_{b}$ in Eq. (27), is defined as

30 $$\gamma \equiv \frac{{\sqrt {1 + 4\tilde{D}/\tilde{\omega }_{b} } - 1}}{{\sqrt {1 + 4\tilde{D}/\tilde{\omega }_{b} } + 1}} = {\text{tanh}}\left( {r_{b} } \right).$$

The other factors in Eqs. (27) and (28) are defined as

31 $${\text{tan}}\left( {2\phi } \right) = 2\overset{\lower0.5em\hbox{$\smash{\scriptscriptstyle\smile}$}}{g} /\left( {\omega_{a} - \overset{\lower0.5em\hbox{$\smash{\scriptscriptstyle\smile}$}}{\omega }_{b} } \right),$$

32 $${\text{tanh}}\left( {2r} \right) = 2\overset{\lower0.5em\hbox{$\smash{\scriptscriptstyle\smile}$}}{g} {\text{cos}}\left( {2\phi } \right)/\left( {\omega_{a} + \overset{\lower0.5em\hbox{$\smash{\scriptscriptstyle\smile}$}}{\omega }_{b} } \right),$$

33 $${\text{tanh}}\left( {2r_{ - } } \right) = \overset{\lower0.5em\hbox{$\smash{\scriptscriptstyle\smile}$}}{g} {\text{sin}}\left( {2\phi } \right)/\epsilon_{ - } ,$$

34 $${\text{tanh}}\left( {2r_{ + } } \right) = - \overset{\lower0.5em\hbox{$\smash{\scriptscriptstyle\smile}$}}{g} {\text{sin}}\left( {2\phi } \right)/\epsilon_{ + } ,$$

where newly defined quantities $\epsilon_{ \pm }$ with a frequency dimension and the eigen frequencies $\Omega_{ \pm }$ are expressed as

35 $$\epsilon_{ \pm } \equiv \sqrt {\frac{{(\omega_{a} + \overset{\lower0.5em\hbox{$\smash{\scriptscriptstyle\smile}$}}{\omega }_{b} )^{2} }}{4} - \overset{\lower0.5em\hbox{$\smash{\scriptscriptstyle\smile}$}}{g}{}^{2} {\text{cos}}^{2} \left( {2\phi } \right)} \pm \sqrt {\frac{{(\omega_{a} - \overset{\lower0.5em\hbox{$\smash{\scriptscriptstyle\smile}$}}{\omega }_{b} )^{2} }}{4} + \overset{\lower0.5em\hbox{$\smash{\scriptscriptstyle\smile}$}}{g}{}^{2} } ,$$

36 $$\Omega_{ \pm } = \sqrt {\epsilon_{ \pm }{}^{2} - g^{2} {\text{sin}}^{2} \left( {2\phi } \right)} .$$

Note that the unitary operator $\hat{U}$ can be rewritten as

37 $$\hat{U} = \hat{U}_{d - } \hat{U}_{d + } \hat{U}_{0} ;$$

that is, a product of $\hat{U}_{0}$ and two one-mode squeezing operators

38 $$\hat{U}_{d \pm } \equiv \hat{U}_{0} \hat{U}_{ \pm } \hat{U}_{0}^{\dagger } = {\text{e}}^{{\left( { - r_{ \pm } /2} \right)\left( {\hat{d}_{ \pm }^{\dagger } \hat{d}_{ \pm }^{\dagger } - \hat{d}_{ \pm } \hat{d}_{ \pm } } \right)}}$$

under a new basis transformed from the original one ($\hat{a}$ and $\hat{b}$) by $\hat{U}_{0}$ as

39 $$\hat{d}_{ - } \equiv \hat{U}_{0} \hat{a}\hat{U}_{0}^{\dagger } ,\;\hat{d}_{ + } \equiv \hat{U}_{0} \hat{b}\hat{U}_{0}^{\dagger } ,$$

In the case of $${\omega }_{a}={\omega }_{b}$$ and the normal phase (zero expectation values of the photonic and atomic fields $$\overline{a }=\overline{b }=0$$) obtained for $$g<\sqrt{{\omega }_{a}{\omega }_{b}}/2$$, we obtain $${r}_{b}=\gamma =0$$, $$\overset{\lower0.5em\hbox{$\smash{\scriptscriptstyle\smile}$}}{\omega }_{b}={\omega }_{b}$$, and $$\overset{\lower0.5em\hbox{$\smash{\scriptscriptstyle\smile}$}}{g}=g$$ from Eqs. (8), (29), and (30). In this case, we can easily find that the ground state $$|0\rangle \propto \widehat{U}|{0}_{a,b}\rangle$$ is an ideal two-mode squeezed vacuum. From Eqs. (31)–(36), under the limit of $${\omega }_{b}\to {\omega }_{a}+{0}^{+}$$, we obtain $${\Omega }_{\pm }=\sqrt{{\omega }_{a}({\omega }_{a}\pm 2g)}$$, $$\phi =-\pi /4$$, $$r=0$$, $$\mathrm{tanh}(2{r}_{-})=-g/({\omega }_{a}-g)$$, and $$\mathrm{tanh}(2{r}_{+})=g/({\omega }_{a}+g)$$. Because the unitary operator $${\widehat{U}}_{0}$$ is simply a superposing operator as $${\widehat{U}}_{0}={\mathrm{e}}^{(\pi /4)({\widehat{a}}^{\dagger}\widehat{b}-{\widehat{b}}^{\dagger}\widehat{a})}$$, the new basis $${\widehat{d}}_{\pm }$$ defined in Eq. (39) is the equal-weight superposition of the original fluctuation operators as $${\widehat{d}}_{\pm }=(\widehat{a}\pm \widehat{b})/\sqrt{2}$$. Then, the ground state is simply expressed as $$|0\rangle \propto \widehat{U}|{0}_{a,b}\rangle ={\widehat{U}}_{d-}{\widehat{U}}_{d+}|{0}_{a,b}\rangle$$; that is, squeezed by $${r}_{\pm }$$ in the two-mode (superposed) basis $${\widehat{d}}_{\pm }$$, and the variances of quadratures defined by $${\widehat{d}}_{-}={\widehat{c}}_{-\pi /\mathrm{4,0}}$$ are obtained as $$(\Delta {X}_{\mathrm{min}}{)}^{2}=(\Delta {X}_{-\pi /\mathrm{4,0},\pi /2}{)}^{2}={\mathrm{e}}^{2{r}_{-}}/4$$ and $$(\Delta {X}_{\mathrm{max}}{)}^{2}=(\Delta {X}_{-\pi /\mathrm{4,0},0}{)}^{2}={\mathrm{e}}^{-2{r}_{-}}/4$$. This is analytical evidence indicating why $$\Delta {X}_{\mathrm{min}}\Delta {X}_{\mathrm{max}}=1/4$$ is satisfied for any $$g$$. When the coupling strength reaches the critical point as $$g\to {\omega }_{a}/2+{0}^{-}$$, the lower eigenfrequency becomes $${\Omega }_{-}\to {0}^{+}$$, and perfect squeezing is obtained as $${r}_{-}\to -\infty$$ in the $${\widehat{d}}_{-}$$ basis. This is analytical evidence indicating why the quadrature variance $$(\Delta {X}_{\mathrm{min}}{)}^{2}$$ vanishes at the SRPT critical point, as demonstrated in Fig. 5.

In the general case with ${\omega }_{a}\ne {\omega }_{b}$ (and in the superradiant phase), we can mathematically confirm that perfect squeezing can be obtained from the expression $|0\rangle \propto \widehat{U}|{0}_{a,b}\rangle$ of the ground state described by the unitary operator $\widehat{U}$ in Eq. (37), while the basis ${\widehat{d}}_{\pm }$ is not a simple superposition of the original fluctuation operators $\widehat{a}$ and $\widehat{b}$, but also includes their creation operators ${\widehat{a}}^{\dagger}$ and ${\widehat{b}}^{\dagger}$. Instead of such a straightforward but complicated analysis, we can also understand perfect squeezing at the SRPT critical point $g=\sqrt{{\omega }_{a}{\omega }_{b}}/2$ as explained in “Discussion”.

## Data Availability

Data sharing is not applicable as no datasets were generated or analyzed during the current study. Code can be provided on request to the corresponding author.
